# Trends in Life Expectancy With Care Needs Based on Long-term Care Insurance Data in Japan

**DOI:** 10.2188/jea.JE20110069

**Published:** 2012-05-05

**Authors:** Rumi Seko, Shuji Hashimoto, Miyuki Kawado, Yoshitaka Murakami, Masayuki Hayashi, Masahiro Kato, Tatsuya Noda, Toshiyuki Ojima, Masato Nagai, Ichiro Tsuji

**Affiliations:** 1 Faculty of Nursing, Fujita Health University School of Health Sciences, Toyoake, Japan; 2 Department of Hygiene, Fujita Health University School of Medicine, Toyoake, Japan; 3 Department of Medical Statistics, Shiga University of Medical Science, Otsu, Japan; 4 Department of Information Science, Fukushima Medical University School of Nursing, Fukushima, Japan; 5 Tsushima Public Health Center, Aichi Prefecture, Tsushima, Japan; 6 Department of Community Health and Preventive Medicine, Hamamatsu University School of Medicine, Hamamatsu, Japan; 7 Division of Epidemiology, Department of Public Health and Forensic Medicine, Tohoku University Graduate School of Medicine, Sendai, Japan

**Keywords:** disability-free life expectancy, life expectancy, care needs, health statistics

## Abstract

**Background:**

Using a previously developed method for calculating expected years of life with care needs based on data from the Japanese long-term care insurance system, we examined recent trends in expected years of life with care needs by age group and prefecture.

**Methods:**

Information on care needs was available from the long-term care insurance system of Japan. Expected years of life with care needs by age group and prefecture in 2005–2009 were calculated.

**Results:**

Expected years of life with care needs at age 65 increased from 1.43 years in 2005 to 1.62 years in 2009 for men, and from 2.99 to 3.44 years for women. As a proportion of total life expectancy, these values show an increase from 7.9% to 8.6% in men and from 12.9% to 14.4% in women. Expected years with care needs did not increase in the age groups of 65 to 69 and 70 to 74 years but markedly increased in the age group of 85 years or older. Expected years with care needs increased in every prefecture during the period studied. The difference in 2005 between the 25th and 75th percentiles in prefectural distributions was 0.16 years for men and 0.35 years for women. The difference remained nearly constant between 2005 and 2009.

**Conclusions:**

Expected number of years of life with care needs increased among Japanese from 2005 to 2009, and there was a wide range in distribution among prefectures. Further studies on coverage of care needs under the long-term insurance program are necessary.

## INTRODUCTION

Life expectancy is a major indicator of population health.^1^ Among the aged population, life expectancy with disability or care needs is important,^2^^,^^3^ as it provides information that is valuable in formulating health policies for elderly adults. Expected years with disability has been evaluated in several countries.^3^^–^^8^

A system of long-term care insurance was recently implemented in Japan,^9^ and a method for calculating expected years of life with and without care needs was developed based on data from this system.^10^^,^^11^ Expected years of life without care needs was calculated and prefectural distributions were reported in previous studies.^11^^,^^12^ However, individuals with care needs were not sufficiently analyzed and recent trends in this population have not been examined.^11^ The recent gain in expected years of total life among adults aged 75 years or older in Japan was greater than that among those aged 65 to 74 years.^13^ The proportion of elderly persons with care needs increases with age.^14^ Thus, it is necessary to analyze recent trends in expected years with care needs by age group.

In the present study, we calculated expected number of years of life with care needs among elderly adults in Japan using a previously developed method based on data from the long-term care insurance system. In addition, we examined trends by age group and prefecture in 2005–2009.

## METHODS

### Long-term care insurance in Japan

The Japanese government implemented mandatory social long-term care insurance on 1 April 2000.^9^^,^^15^ Every adult aged 65 years or older in Japan is eligible. Level of care need is based on the individual’s physical and mental status, as evaluated by the insurance system. The level determines the extent of service coverage.

### Data

We used Japanese population, mortality, and life table data from 2005–2009.^13^^,^^16^^,^^17^ Excepting life tables, data were available from all 47 prefectures. Data on care needs were obtained from the *Report on Long-Term Care Insurance Services* and the *Survey of Long-Term Care Benefit Expenditures* at the end of each September from 2005 to 2009.^14^^,^^17^^,^^18^ The former report is based on administrative records of the long-term care insurance system and includes the actual number of persons for each care need level, as certified by the insurance system, in the age groups of 65 to 74 and 75 years or older in all prefectures. However, it does not include separate values for men and women. The latter survey is based on long-term care benefit statements and includes the approximate number of persons for care need level in 5-year age bands among men and women of all prefectures. Values were estimated using the totals of the actual numbers multiplied by the proportions of the approximate numbers.

### Calculation of expected years with care needs

We calculated expected number of years with care needs by using the previously developed method, based on the abovementioned data, as follows.^3^^,^^11^ Care needs for persons aged 65 or older were evaluated using the care need levels certified by the long-term care insurance system of Japan.^15^ A level 2 or greater care need was classified in the present study as “having care needs”; all other care-need levels were classified as “no care needs” in our analysis. Sex- and age-specific prevalences of persons with care needs were then calculated for each prefecture in 2005–2009. The age groups were 65 to 69, 70 to 74, 75 to 79, 80 to 84, and 85 years or older.

Using the Sullivan method,^19^ we calculated expected number of years with care needs at age *x* years during the interval between age *y* and age *z* as follows:

$$\Sigma \pi_{i}L_{i}/l_{x}$$

where Σ represents the sum between *y* and *z* years in age group *i*, π*_i_* is the age-specific prevalence of care needs, *L_i_* is the stationary population, and *l_x_* is the number of survivors in the life table. The underlying assumption in this calculation is that age-specific prevalence of care needs in the stationary population is equivalent to that observed in the real population.^3^ Data from Japanese nationwide life tables were available. Life tables for prefectures were constructed using Chiang’s method, based on prefectural death rates.^20^

## RESULTS

**Figure 1.**
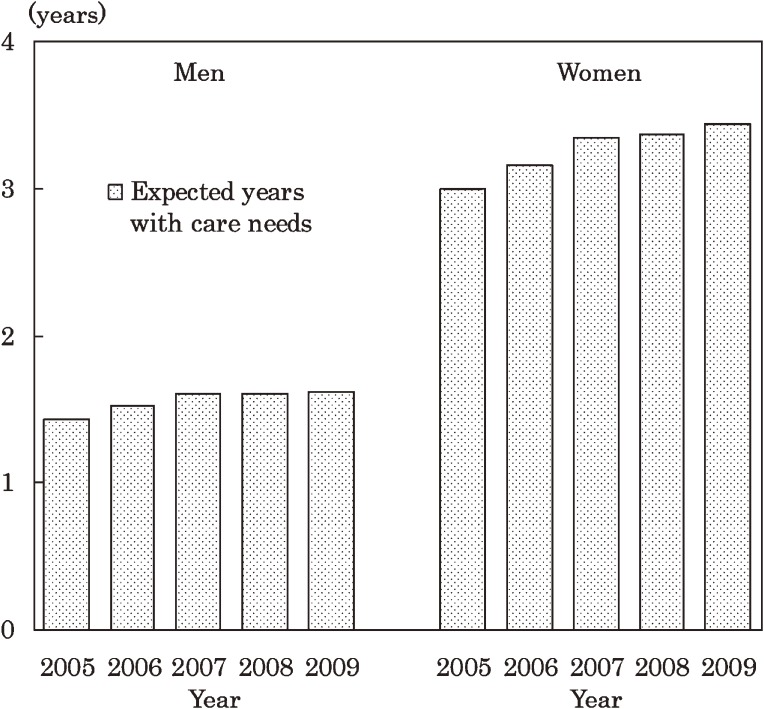
Expected number of years with care needs at age 65 among Japanese men and women in 2005–2009

Figure 1 shows expected years of life with care needs at age 65 years for men and women in 2005–2009. Expected years of life with care needs at age 65 for men was 1.43 in 2005, and monotonically increased to 1.63 in 2009. The values for women monotonically increased from 2.99 to 3.44 during the same period.

Table 1 shows total life expectancy and expected years of life with care needs at age 65 in men and women in 2005 and 2009, by age group. The proportion of expected years with care needs to total life expectancy at age 65 for men was 7.9% in 2005 and 8.6% in 2009. The corresponding proportions for women were 12.9% and 14.4%.

**Table 1.** Total life expectancy and expected years of life with care needs at age 65 by age group among Japanese men and women in 2005 and 2009

Sex	Age group (years)	2005		2009	
	
		Life expectancy (years)	Expected years with care needs (years)	Life expectancy (years)	Expected years with care needs (years)
Men	65–69	4.82	0.09 (1.8)	4.83	0.09 (1.8)
	70–74	4.35	0.16 (3.7)	4.41	0.16 (3.7)
	75–79	3.65	0.24 (6.5)	3.78	0.26 (6.8)
	80–84	2.71	0.30 (11.3)	2.89	0.35 (12.1)
	85+	2.58	0.64 (24.8)	2.96	0.77 (25.9)
	Total	18.11	1.43 (7.9)	18.88	1.62 (8.6)

Women	65–69	4.92	0.07 (1.3)	4.93	0.07 (1.3)
	70–74	4.71	0.14 (3.0)	4.75	0.14 (3.0)
	75–79	4.36	0.28 (6.5)	4.44	0.31 (6.9)
	80–84	3.78	0.54 (14.4)	3.91	0.59 (15.0)
	85+	5.38	1.96 (36.4)	5.95	2.34 (39.4)
	Total	23.16	2.99 (12.9)	23.97	3.44 (14.4)

Number of expected years with care needs as a proportion of life expectancy is shown as a percentage in parentheses.

Among men, expected number of years with care needs in 2005 increased from 0.09 years for the age group of 65 to 69 years to 0.64 years for the age group of 85 years or older. The numbers for women in 2005 and for men and women in 2009 also increased with advancing age. The difference between 2005 and 2009 was less than 0.01 years for the age groups of 65 to 69 and 70 to 74 years, less than 0.05 years for the age groups of 75 to 79 and 80 to 84 years, and 0.13 years for men and 0.38 years for women for the age group of 85 years or older.

**Figure 2.**
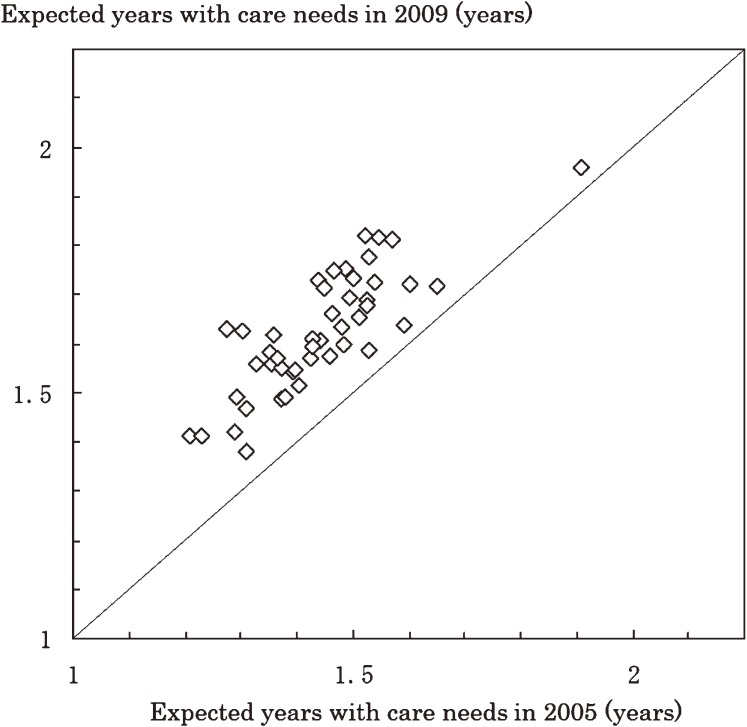
Expected number of years with care needs at age 65 among men for all Japanese prefectures in 2005 and 2009

**Figure 3.**
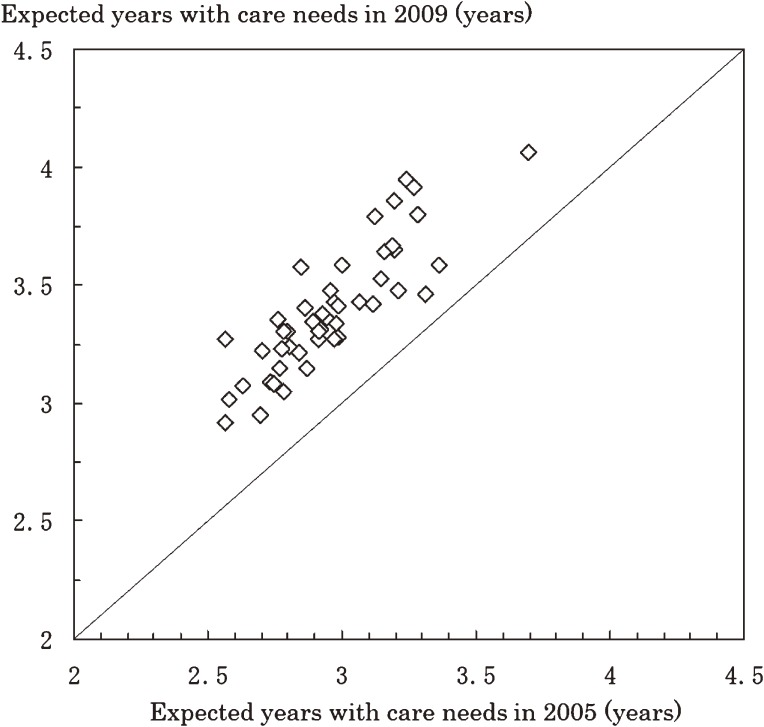
Expected number of years with care needs at age 65 among women for all Japanese prefectures in 2005 and 2009

Expected years with care needs at age 65 years in 2005 and 2009, by prefecture, are shown in Figures 2 and 3 for men and women, respectively. The expected years with care needs for men and women increased in every prefecture from 2005 to 2009. Number of expected years with care needs in 2009 between 47 prefectures ranged from 1.38 to 1.96 among men and from 2.92 to 4.06 among women.

Table 2 shows prefectural distributions of total life expectancy and expected years with care needs at age 65 years among men and women in 2005 and 2009. The 25th and 75th percentiles of expected years with care needs at age 65 years were 1.36 and 1.52 years, respectively, for men in 2005. The difference between these percentiles was 0.16 years for men and 0.35 years for women in 2005, and 0.16 years for men and 0.32 years for women in 2009.

**Table 2.** Prefectural distributions of total life expectancy and expected years of life with care needs at age 65 for Japanese men and women in 2005 and 2009

Sex		Prefectural distribution in 2005			Prefectural distribution in 2009		
		
		Percentiles		Difference^a^	Percentiles		Difference^a^
		
		25th	75th		25th	75th	
Men	Life expectancy (years)	17.95	18.30	0.35	18.58	19.03	0.45
	Expected years with care needs (years)	1.36	1.52	0.16	1.55	1.71	0.16

Women	Life expectancy (years)	22.92	23.60	0.68	23.64	24.20	0.56
	Expected years with care needs (years)	2.78	3.14	0.35	3.23	3.55	0.32

^a^Difference between 25th and 75th percentiles.

## DISCUSSION

Expected years of life with care needs at age 65 years increased in 2005–2009, as did the proportion of those years to total life expectancy. These results indicate that the duration of senior life with disabilities increased in the Japanese population. An increase in the number of expected years of life with a light or moderate disability to total life (ie, including younger lives) was reported for 1995–2004 in the Japanese population.^8^ Prolongation of expected years with disability has been reported in some countries, while a decrease has been noted in others.^3^^–^^6^

We observed temporal trends in expected years with care needs at age 65 by age group. Those years did not increase in 2005–2009 in the age groups of 65 to 69 and 70 to 74 years; however, they markedly increased in the age group of 85 years or older. Recently, life expectancy in the age groups of 65 to 69 and 70 to 74 years is very high in Japan.^13^ Because recent gains in expected years of total life were very small (Table 1), however, the absence of an increase in those with care needs in these age groups would not be surprising. Nevertheless, there were some gains in expected years of total life in the age group of 85 years or older. The gains in expected years of relatively older life (eg, age >90 years) would lead to an increase in those with care needs in the age group of 85 years or older.

Expected years of life with care needs at age 65 years increased in 2005–2009 in every prefecture. The differences between the 25th and 75th percentiles in prefectural distributions was 0.16 years for men and 0.35 years for women in 2005. Those differences remained virtually constant between 2005 and 2009. Disparities in expected years with care needs or disability by geographic area have been reported in several reports.^3^^,^^11^^,^^12^^,^^21^^,^^22^

There are many factors related to mortality and care needs in elderly people. Correspondingly, many factors influence temporal trends and prefectural differences in expected years with care needs observed in the present study. There have been influential studies of these factors that used correlation analysis of prefectural data in Japan. One report found that, among 181 factors related to demographic, socioeconomic status, health status and behavior, medical environment, social relationships, climate, and other areas, 3 factors were associated with long disability-free life expectancy: good self-reported health status, a high proportion of older workers, and the presence of a large number of public health nurses.^23^ Another report observed that expected years with disability at age 65 years was negatively correlated with the rate of elderly adults living with a son or daughter (among men), the residential capacity of institutes for the elderly (among women), and the availability of care services (among men and women).^24^ A third study reported that disability-adjusted life expectancy at age 65 years was correlated with the overall unemployment rate.^25^ Other, similar ecological studies found that disability-free life expectancy was associated with illiteracy rate and the proportion of smokers (in Spain), with social class (in England), and with economic status (in China).^22^^,^^26^^,^^27^ Prospective studies of persons aged 65 years or older indicated that active life expectancy was associated with level of education, smoking status, and physical activity.^28^^,^^29^ These findings confirmed that several factors, including socioeconomic status, are related to temporal trends and prefectural differences in expected years with care needs, as observed in the present study. Further studies of determinants are warranted.

There were some limitations and problems in the present study. We used Japanese long-term care insurance data, which have been used to estimate disability-free life expectancy in several studies.^10^^–^^12^^,^^30^ Our findings could be affected by changes in the long-term care insurance system. Increased insurance coverage of care needs would lead to incorrect higher estimates of expected years with care needs. However, it was reported that applications for insurance rapidly improved during the first 3 years after introduction of the system and that coverage of care needs in a ward in Sendai City in 2002 was nearly complete.^31^ The coverage of care needs in 2005 should therefore be sufficiently high and stable to accurately estimate expected years with care needs of elderly adults in Japan.^11^ The insurance system underwent a major change when new preventive benefits were introduced in 2006.^15^ The goal of these benefits is to prevent seniors from becoming dependent. However, the target includes only seniors with lesser needs, not those with a care need level of 2 or more, ie, those who were classified as having care needs in our study. Information on coverage of care needs under the insurance system would not be sufficient for appropriate evaluation of temporal trends in expected years with care needs.

We used actual numbers of persons with care needs from the *Report on Long-Term Care Insurance Services* and sex- and age-specific proportions of approximate numbers of persons with care needs from the *Survey of Long-term Care Benefit Expenditures*.^14^^,^^18^ When using only those approximate numbers, as in another study, expected years with care needs at age 65 slightly changed^11^: 1.44 years for men and 3.03 years for women in 2005, and 1.65 years for men and 3.49 years for women in 2009 (the respective values in Table 1 were 1.43, 2.99, 1.62, and 3.44 years).

As required by the previously developed method used in the present study, we classified care need levels of 2 or higher as having care needs, and other levels as having no care needs.^11^ A previous report indicated that many public health workers had accepted this classification for calculating expected years of life with and without care needs.^32^ In addition, we used the Sullivan method for calculating expected years with care needs. Although it is assumed that age-specific prevalence of care needs in the stationary population is equivalent to that in the real population, this method is a common tool for estimating disability-free life expectancy based on cross-sectional data on disability.^3^^,^^19^ Life-table data for all of Japan were available.^13^ Life tables in prefectures were constructed using Chiang’s method, based on prefectural death rates. Chiang’s method is a standard technique for constructing an abridged life table.^11^^,^^20^ We observed expected years with care needs in 2005–2009. When evaluating such trends, a longer observation period might be more useful. We hope that future reports of official statistics will include such information on expected years with care needs.

In conclusion, expected years of life with care needs increased among Japanese from 2005 to 2009, although there was a wide range in prefectural distributions. Further studies on coverage of care needs under the long-term insurance program are necessary to confirm these findings.
